# Research Progress on Signalling Pathways Related to Sepsis-Associated Acute Kidney Injury in Children

**DOI:** 10.3390/cimb47110888

**Published:** 2025-10-27

**Authors:** Zhenkun Zhang, Meijun Sheng, Yiyao Bao, Chao Tang

**Affiliations:** Children’s Hospital, Zhejiang University School of Medicine, National Clinical Research Center for Child Health, 3333 Binsheng Road, Hangzhou 310052, China; 6514147@zju.edu.cn (Z.Z.); smj512@zju.edu.cn (M.S.)

**Keywords:** acute kidney injury, biomarkers, inflammation, metabolic reprogramming, pyroptosis, pediatric sepsis, signalling pathways, therapeutic targets

## Abstract

Sepsis-associated acute kidney injury (SA-AKI) is a prevalent and life-threatening complication in critically ill children, contributing to high mortality rates (up to 30%) and long-term renal dysfunction in pediatric intensive care units. This review synthesizes recent advances in the signalling pathways underlying SA-AKI, emphasizing pediatric-specific mechanisms, biomarkers, and therapeutic targets. This review covers inflammatory cascades via TLR/NF-κB leading to cytokine storms (IL-6, TNF-α); apoptosis and necrosis involving mitochondrial Bcl-2 dysregulation and OLFM4; and emerging processes like pyroptosis (NF-κB-mediated), metabolic reprogramming (choline deficiency and Nrf2-mitophagy), and novel routes such as cGAS-STING and TGF-β signalling. Biomarkers like urinary OLFM4, DKK3, NGAL, and serum suPAR, alanine, and Penkid enable early diagnosis and risk stratification, with models like PERSEVERE-II enhancing prognostic accuracy. Therapeutic strategies include fluid optimization, renal replacement therapies (CRRT, SLED-f), and pathway-targeted interventions such as choline supplementation, oXiris for cytokine removal, Humanin for immunomodulation, and investigational cGAS-STING inhibitors. Despite progress, challenges persist in translating animal models to pediatric trials and addressing heterogeneity. Integrating multi-omics and precision medicine holds promise for improving outcomes, underscoring the need for multicenter studies in children.

## 1. Introduction

SA-AKI represents a critical and multifaceted complication in pediatric critical care, characterized by a sudden decline in renal function amidst systemic inflammation triggered by infection. Defined by consensus criteria such as the Kidney Disease: Improving Global Outcomes (KDIGO) or pediatric-specific adaptations like pRIFLE (pediatric Risk, Injury, Failure, Loss, End-stage kidney disease), SA-AKI manifests through elevated serum creatinine, reduced urine output, and electrolyte imbalances, often exacerbating multi-organ dysfunction syndrome (MODS) in children [1,2]. In pediatric populations, SA-AKI differs from adult cases due to immature renal physiology, higher baseline glomerular filtration rates, and greater susceptibility to hemodynamic instability, making early recognition and intervention paramount [3]. The interplay between sepsis-induced systemic responses and renal vulnerability underscores the urgency of understanding its pathophysiology, as untreated SA-AKI can lead to chronic kidney disease (CKD) or end-stage renal disease (ESRD) in survivors [4].

Epidemiologically, SA-AKI is alarmingly prevalent among critically ill children, with incidence rates ranging from 12% to 50% in pediatric intensive care units (PICUs) worldwide, particularly in resource-limited settings [5,6]. Recent meta-analyses indicate a pooled in-hospital mortality rate of approximately 18.27% among hospitalized children with AKI, with sepsis as the leading aetiology in up to 46.5% of cases [7,8]. In septic shock scenarios, the risk escalates, with studies reporting AKI occurrence in over 70% of affected children, driven by factors such as older age (adolescents at higher risk), lower baseline estimated glomerular filtration rate (eGFR), and mechanical ventilation dependency [9,10]. For instance, a retrospective cohort in a Taiwanese PICU revealed that sepsis contributed to 46.5% of AKI cases, with a mortality rate of 44.2%, highlighting prognostic factors like prolonged hypotension and nephrotoxic drug exposure [11]. While regional studies like this one highlight the high incidence and specific challenges in high-resource contexts, the global burden of pediatric SA-AKI is profoundly impacted by socio-economic factors. Specifically, the disease severity and associated mortality are notably higher in low- and middle-income countries (LMICs), primarily due to prolonged diagnostic delays and restricted availability of essential treatments like renal replacement therapy (RRT) [12]. Similarly, in cardiac intensive care units, AKI prevalence reaches 30–40%, associated with post-surgical sepsis and augmented renal clearance in younger patients [13,14].

The clinical impact of SA-AKI extends beyond acute mortality, influencing long-term outcomes such as prolonged PICU stays, increased healthcare costs, and persistent renal impairment. Survivors often face a 2–3-fold heightened risk of CKD, with studies linking severe AKI (KDIGO stage 2–3) to reduced quality of life and neurodevelopmental delays in children [15,16]. Risk factors include sepsis severity (e.g., septic shock), underlying comorbidities like hypertension or congenital heart disease, and iatrogenic elements such as vasoactive agents or antibiotics [17,18]. Prognostic models incorporating biomarkers like serum creatinine, lactate dehydrogenase (LDH), and antithrombin III (AT3) have shown promise in predicting severe AKI and mortality, with areas under the receiver operating characteristic curve (AUC) exceeding 0.8 [19].

Traditionally, SA-AKI was attributed primarily to renal hypoperfusion from septic shock, leading to acute tubular necrosis (ATN). However, contemporary research has unveiled a more intricate pathogenesis involving dysregulated signalling pathways that amplify inflammation, oxidative stress, and cellular death [20]. Key inflammatory cascades, such as Toll-like receptor (TLR)/nuclear factor-kappa B (NF-κB), drive cytokine storms (e.g., IL-6, TNF-α), exacerbating endothelial dysfunction and tubular injury [21]. Apoptosis and necrosis pathways, mediated by mitochondrial Bcl-2 family proteins and olfactomedin-4 (OLFM4), contribute to renal cell loss, while metabolic derangements like choline deficiency impair energy homeostasis [22]. Emerging mechanisms include pyroptosis via NF-κB/inflammasome activation, which promotes gasdermin-D-dependent pore formation and cytokine release in renal tubular cells [23,24]. The cyclic GMP-AMP synthase-stimulator of interferon genes (cGAS-STING) pathway, activated by mitochondrial DNA leakage, amplifies type I interferon responses and inflammation, linking innate immunity to AKI progression [25,26]. Additionally, transforming growth factor-beta (TGF-β) signalling fosters fibrosis and epithelial–mesenchymal transition, potentially bridging acute injury to chronic sequelae [27,28]. These pathways are particularly relevant in pediatrics, where developmental immaturity heightens vulnerability to microbial DNA sensing and inflammatory amplification [29].

Despite advances, significant research gaps persist. Pediatric-specific data remain limited compared to adults, with most mechanistic insights derived from animal models or adult cohorts, potentially overlooking age-dependent renal maturation and immune responses [30]. Diagnostic heterogeneity across criteria (e.g., pRIFLE vs. KDIGO) complicates incidence comparisons, and translational barriers hinder the application of novel therapeutics like cGAS-STING inhibitors in children [31,32]. Moreover, integrating multi-omics approaches (e.g., metabolomics for choline pathways) with clinical biomarkers is underdeveloped, impeding precision medicine [33].

This systematic review aims to synthesize recent progress (2015–2025) in SA-AKI signalling pathways, emphasizing pediatric epidemiology, mechanisms, biomarkers, and therapeutic targets. We seek to bridge knowledge gaps and inform future multidisciplinary research for improved pediatric outcomes by addressing these elements.

## 2. Epidemiology and Clinical Risk Factors

SA-AKI is a prevalent complication in critically ill children, particularly in pediatric intensive care units (PICUs), significantly contributing to morbidity and mortality. The epidemiology of SA-AKI reveals a broad incidence range, influenced by diagnostic criteria, study settings, and regional variations. Studies report SA-AKI affecting 14% to 87% of children with sepsis, with a pooled incidence of approximately 25–75% in critically ill cohorts [34,35]. A systematic review of 47 studies noted that varying definitions contribute to this range, underscoring the need for standardized reporting [36]. In PICUs, sepsis is linked to AKI in 10–55% of cases, with higher rates in severe sepsis or septic shock [35,37]. A bicentric study found a 16% AKI incidence overall, with septic AKI forming a substantial subset [38]. Globally, sepsis accounts for 8% of PICU admissions, with shock and sepsis triggering AKI in up to 46.5% of cases [39,40].

Clinical manifestations of SA-AKI are diverse and often insidious, complicating early detection. Common signs include oliguria or anuria (urine output < 0.5 mL/kg/h), elevated serum creatinine, fluid overload, and electrolyte imbalances like hyperkalemia or hyponatremia [41,42]. These symptoms typically follow initial sepsis indicators such as fever, tachycardia, or hypotension and may coincide with multi-organ dysfunction syndrome (MODS) [43]. In neonates and young infants, manifestations are subtler due to immature renal function, often presenting as non-oliguric AKI, which is frequently overlooked [44]. Diagnostic criteria are critical for identification. The pediatric Risk, Injury, Failure, Loss, End-stage (pRIFLE) criteria stratify AKI based on estimated creatinine clearance (eCrCl) changes and urine output, ranging from Risk (eCrCl decrease ≥ 25%) to failure (eCrCl decrease ≥ 75% or urine output < 0.3 mL/kg/h for 24 h) [45]. The Kidney Disease: Improving Global Outcomes (KDIGO) criteria, adapted for pediatrics, use absolute serum creatinine rises (≥0.3 mg/dL within 48 h) or relative increases (≥1.5 times baseline) alongside urine output thresholds [46]. Comparative studies show KDIGO detects AKI in 67.7% of septic children, while pRIFLE identifies 74.2%, both correlating with poor outcomes but differing in sensitivity [47]. Challenges persist in neonates due to dynamic baseline creatinine [48].

**Figure 1:**
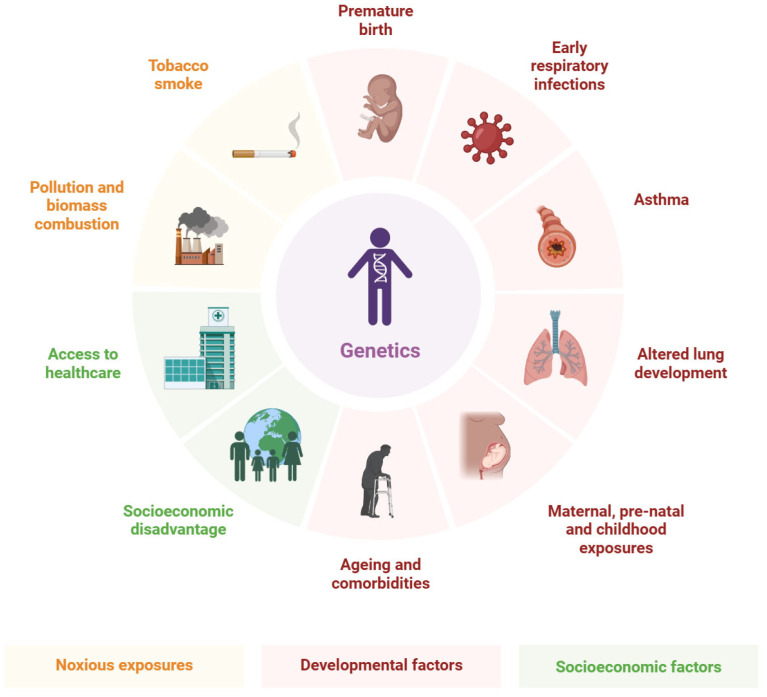
Epidemiology and Clinical Risk Factors.

Major risk factors for SA-AKI are multifactorial, encompassing patient-specific, sepsis-related, and iatrogenic elements. Sepsis severity, particularly septic shock, is a primary driver, causing hypoperfusion and endothelial damage [49]. Older age (e.g., adolescents) and lower baseline eGFR independently predict SA-AKI, with odds ratios indicating heightened vulnerability in school-aged children [50]. Mechanical ventilation is a significant risk, associated with AKI in up to 62.6% of septic cases due to altered renal hemodynamics [51]. Nephrotoxic exposures, including antibiotics (e.g., vancomycin, aminoglycosides) and vasoactive drugs, contribute to 45–46.8% of cases [52,53]. Hypovolemic shock, hypertension, and underlying conditions like congenital heart disease or glomerulonephritis amplify risks, with respiratory failure and ileus as common hospital-acquired factors [54]. Obesity has emerged as a modifier, increasing early SA-AKI risk via inflammation [55]. Infections and dehydration predominate in low-resource settings, reflecting socioeconomic influences [56], as shown in Table 1 and Figure 1.

The prognostic impact of SA-AKI is profound, with affected children facing 18–60% mortality, prolonged PICU stays, and increased disability [57]. SA-AKI doubles the risk of death or new moderate disability, with KDIGO stage 2–3 linked to worse outcomes [58]. Hospital mortality reaches 18–44.2% in septic AKI cohorts, higher than non-AKI sepsis (57–60% in severe cases) [59,60]. Long-term sequelae include a 2–3-fold increased risk of chronic kidney disease (CKD) and neurodevelopmental impairments, reducing quality of life [61,62]. Phenotypic variations, such as late versus early AKI, worsen prognosis, emphasizing the need for risk stratification [63]. In resource-limited areas, outcomes are poorer due to delayed renal replacement therapy (RRT) access [64].

In summary, SA-AKI’s epidemiology underscores a high burden in pediatric sepsis, driven by identifiable risks and linked to dire outcomes. Early application of pRIFLE/KDIGO criteria is crucial for mitigation [45,46].

## 3. Pathophysiology and Key Signalling Pathways of SA-AKI

SA-AKI in children arises from a complex interplay of systemic inflammation, hemodynamic instability, and direct renal cellular damage, diverging from the traditional view of mere hypoperfusion leading to acute tubular necrosis (ATN). In pediatric patients, the immature renal system—characterized by lower glomerular filtration rates in neonates and heightened sensitivity to cytokines—amplifies vulnerability to sepsis-induced insults [65,66]. Pathophysiologically, SA-AKI involves endothelial dysfunction, microvascular thrombosis, and tubular epithelial cell injury, driven by pathogen-associated molecular patterns (PAMPs) and damage-associated molecular patterns (DAMPs) that activate innate immune responses [67,68]. This results in a cytokine storm, oxidative stress, and metabolic shifts, progressing to multi-organ failure if unchecked. Recent research emphasizes programmed cell death modalities and novel signalling cascades, offering insights into pediatric-specific mechanisms where rapid renal development influences pathway dynamics [69,70]. Understanding these pathways is crucial for targeted therapies, as SA-AKI in children often resolves faster than in adults but carries long-term risks like chronic kidney disease (CKD) [71].

### 3.1. Inflammatory Signalling Pathways

**Figure 2:**
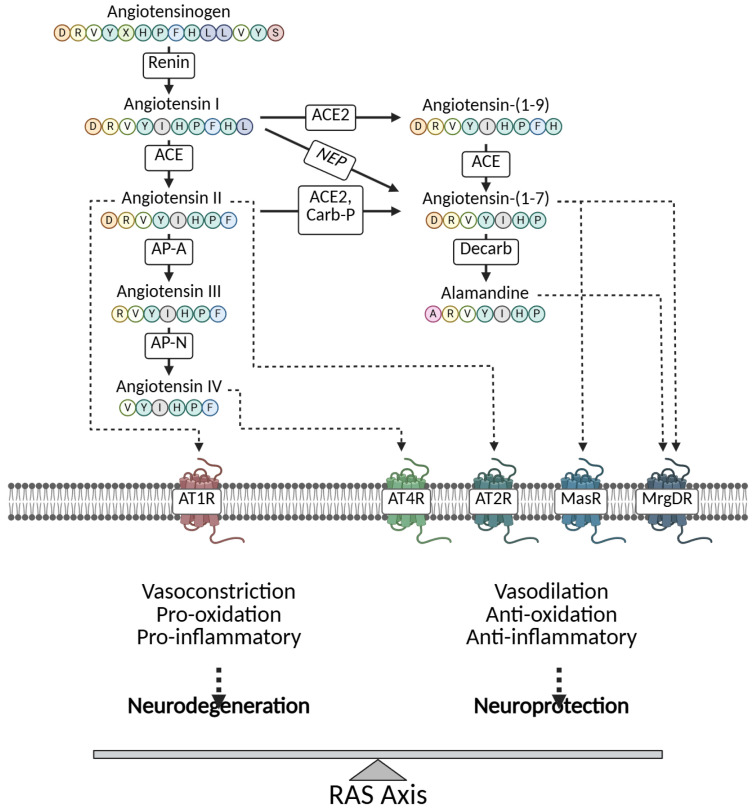
Pathophysiology and key signalling pathways of SA-AKI.

Inflammation is a cornerstone of SA-AKI pathogenesis, initiated by Toll-like receptors (TLRs) recognizing PAMPs and DAMPs, leading to nuclear factor-kappa B (NF-κB) activation and cytokine release [72,73]. In pediatric SA-AKI, TLR4, expressed on renal tubular epithelial cells (RTECs) and endothelial cells, binds lipopolysaccharide (LPS) from Gram-negative bacteria, triggering MyD88-dependent signalling that phosphorylates NF-κB, promoting transcription of pro-inflammatory cytokines like interleukin-6 (IL-6) and tumour necrosis factor-alpha (TNF-α) [74,75]. Elevated IL-6 and TNF-α correlate with AKI severity in septic children, exacerbating tubular injury and endothelial permeability [76,77]. MicroRNAs (miRNAs) like miR-16-5p modulate this pathway, upregulating in RTECs to enhance apoptosis and inflammation via NF-κB [78,79]. Pediatric studies show that inhibiting TLR/NF-κB with antioxidants reduces cytokine storms and improves outcomes in sepsis models [80,81]. Crosstalk with Nrf2 pathways further regulates inflammation, where NF-κB suppression activates Nrf2 for antioxidant defence [82], as shown in Figure 2. The revised figure illustrates not only the classical inflammatory cascades but also includes emerging regulatory mechanisms discussed in Section 3.1. Specifically, the figure now depicts the activation of the Nrf2 pathway as an endogenous anti-inflammatory/antioxidant response and the role of non-coding RNAs (e.g., miR-16-5p) in modulating key targets within the inflammatory signalling networks.

### 3.2. Apoptosis and Necrosis-Related Signals

Apoptosis and necrosis dominate cellular death in SA-AKI, with mitochondrial pathways central in pediatric contexts due to the high metabolic demands of developing kidneys [83,84]. The Bcl-2 family regulates mitochondrial outer membrane permeabilization (MOMP), where pro-apoptotic Bax/Bak oligomerize to release cytochrome c, activating caspases-9/3 for apoptosis [85,86]. In septic children, OLFM4, a neutrophil granule protein, promotes RTEC apoptosis; juvenile OLFM4-null mice show sepsis protection, suggesting OLFM4 as a pediatric biomarker [87,88]. Necrosis, often regulated, involves receptor-interacting protein kinase 3 (RIPK3) in necroptosis, amplifying inflammation via DAMPs [89]. Emerging in pediatrics is pyroptosis, a lytic death form via NLRP3 inflammasome, caspase-1, and gasdermin-D (GSDMD), releasing IL-1β/IL-18 [90,91]. NF-κB mediates pyroptosis in SA-AKI, with pediatric models showing NLRP3 inhibition reduces tubular damage [92,93]. These processes interact, with apoptosis shifting to necroptosis under caspase inhibition, worsening pediatric outcomes [94].

The role of apoptosis in SA-AKI, particularly in the pediatric population, is increasingly viewed through a dual, context-dependent lens, determined by its timing, extent, and mode. While excessive or late-stage apoptosis, frequently accompanied by a shift toward uncontrolled necrosis or necroptosis, is clearly detrimental and worsens outcomes, controlled and early-stage apoptosis may be protective. This regulated cell death enables the swift and clean clearance of irreparably damaged renal tubular epithelial cells (RTECs) without releasing inflammatory Damage-Associated Molecular Patterns (DAMPs) into the kidney microenvironment, thus potentially curtailing the inflammatory cascade, as the recent literature suggests [95]. However, when the rate of cell death exceeds the kidney’s clearance and repair capacity, the process becomes overwhelmingly detrimental, leading to structural and functional collapse. Therefore, therapeutic strategies should aim not just to block apoptosis entirely, but to modulate the balance toward this beneficial, controlled cell clearance mechanism.

Beyond the traditionally recognized forms of cell death, ferroptosis and NETosis are emerging as significant contributors to the pathogenesis of pediatric SA-AKI, linking oxidative stress, lipid metabolism, and immunothrombosis. Ferroptosis, a form of regulated necrotic cell death driven by iron-dependent lipid peroxidation and resulting in severe oxidative stress, is increasingly implicated in renal tubular epithelial cell (RTEC) injury during sepsis. The resulting toxic lipid reactive oxygen species (ROS) compromise mitochondrial function and initiate cell death [96]. Similarly, NETosis—the active release of neutrophil extracellular traps (NETs) composed of chromatin, histones, and granular proteins—plays a complex, often detrimental role. While NETs are essential for pathogen containment, excessive formation contributes to microvascular thrombosis, directly causes RTEC damage via cytotoxic histones, and fuels the inflammatory cascade, thus worsening SA-AKI outcomes, particularly in the microcirculation of the pediatric kidney [97].

### 3.3. Metabolic Abnormality Pathways

Metabolic reprogramming in SA-AKI disrupts renal energy homeostasis, particularly in energy-intensive RTECs [98]. Choline metabolism is altered in pediatric SA-AKI, with deficiency impairing phospholipid synthesis and mitochondrial function; supplementation in murine models attenuates injury by restoring energy metabolism [99]. Nrf2, a transcription factor, orchestrates antioxidant responses; in SA-AKI, Nrf2 activation via Keap1 dissociation induces heme oxygenase-1 (HO-1) and NADPH quinone oxidoreductase-1 (NQO1), mitigating oxidative stress [100]. Mitophagy, selective mitochondrial autophagy, is regulated by Nrf2; the PINK1/Parkin pathway clears damaged mitochondria, preventing apoptosis in septic RTECs [101,102]. Pediatric studies show defective mitophagy in sepsis, with Nrf2 agonists enhancing clearance and reducing AKI severity [103,104]. Metabolic shifts to glycolysis (Warburg effect) occur, but persistent impairment leads to fibrosis [105,106].

The intense inflammatory state in SA-AKI is inextricably linked to profound metabolic reprogramming within renal cells, moving beyond mere hypoperfusion effects. This critical abnormality serves as a key driver of RTEC dysfunction. Specifically, inflammation triggers significant mitochondrial dysfunction, characterized by decreased oxygen consumption rates, impaired oxidative phosphorylation (OXPHOS), and reduced ATP generation, leading to an “energy crisis” in the kidney. Simultaneously, many immune and tubular cells undergo a shift towards aerobic glycolysis (the Warburg effect), even in the presence of sufficient oxygen, diverting metabolic intermediates away from energy production and towards biomass accumulation and inflammatory signalling. Furthermore, sepsis significantly disrupts amino acid metabolism (e.g., glutamine and arginine), essential substrates for renal energy homeostasis and antioxidant defence systems. This systemic and cellular metabolic collapse, driven by inflammatory signals like NF-κB, exacerbates cell injury and significantly contributes to the persistent functional decline characteristic of pediatric SA-AKI.

### 3.4. Immune Pathways

Immune dysregulation in SA-AKI involves complement activation and post-translational modifications (PTMs) amplifying inflammation [107,108]. Complement pathways (classical, alternative, lectin) converge on C3 convertase, generating C3a/C5a anaphylatoxins that recruit neutrophils and promote cytokine release in RTECs [109,110]. In pediatric SA-AKI, urinary complement fragments correlate with severity, with C5a inhibition reducing tubular damage [111,112]. PTMs like phosphorylation (on NF-κB) and ubiquitination (on Keap1 for Nrf2 activation) regulate immune responses; in sepsis, dysregulated PTMs enhance inflammasome assembly [39,113]. Pediatric data show complement-targeted therapies mitigate AKI progression [114,115].

### 3.5. Emerging Pathways

Emerging pathways like cGAS-STING and TGF-β offer new insights into SA-AKI [116,117]. cGAS detects cytosolic DNA, synthesizing cGAMP to activate STING, inducing type I interferons and NF-κB; in SA-AKI, mitochondrial DNA leakage activates this, promoting inflammation [118,119]. Pediatric models show STING inhibition reduces RTEC pyroptosis [120,121]. TGF-β signalling drives fibrosis via Smad3, linking acute inflammation to CKD; in children, elevated TGF-β correlates with poor recovery [122,123]. STING-PERK pathway, a non-canonical branch, regulates ER stress and mitophagy, with dysregulation exacerbating AKI [124,125]. These pathways represent therapeutic targets, with inhibitors promising in models [126,127] (Table 2).

### 3.6. The Influence of Genetics and Epigenetics on SA-AKI Heterogeneity

The notable heterogeneity in pediatric SA-AKI incidence and outcomes suggests that intrinsic host factors, beyond standard clinical variables, play a crucial role. Specifically, genetic and epigenetic variations are emerging as key determinants influencing an individual child’s susceptibility and response to sepsis-induced kidney injury. Genetic polymorphisms in genes encoding pattern recognition receptors (e.g., TLRs), inflammatory cytokines (e.g., IL-6, TNF-α), and components of the coagulation cascade can modulate the intensity and duration of the signalling cascades discussed in this review, thereby contributing to disease heterogeneity. Furthermore, epigenetic mechanisms—including DNA methylation, histone modification, and non-coding RNAs (ncRNAs) such as microRNAs—are recognized as dynamic regulators of renal cells under stress. These epigenetic changes can alter the expression of key signalling molecules (e.g., NF-κB, p53) in response to septic insults, offering a refined layer of control over the SA-AKI trajectory. Integrating these genetic and epigenetic insights will be vital for future biomarker development and advancing precision medicine approaches.

## 4. Biomarkers

Biomarkers are pivotal for the early diagnosis, risk stratification, and prognosis of SA-AKI in pediatric patients, enabling timely interventions to mitigate renal damage and improve outcomes. In children, biomarkers must account for developmental variations in renal function, differing from adult profiles due to immature glomerular filtration and tubular reabsorption [65,66]. This section reviews established and emerging biomarkers, categorized as urinary and blood-based, alongside risk stratification models like PERSEVERE-II, focusing on their clinical utility in early detection and prognostic assessment. Table 3 from recent studies summarizes their performance, including sensitivity, specificity, and area under the receiver operating characteristic curve (AUC).

### 4.1. Urinary Biomarkers

Urinary biomarkers are non-invasive indicators of renal tubular injury, offering high sensitivity for early SA-AKI detection. Neutrophil gelatinase-associated lipocalin (NGAL) is a 25 kDa protein upregulated in renal tubular epithelial cells (RTECs) during stress, detectable within hours of injury [67,148]. In pediatric SA-AKI, urinary NGAL levels rise significantly, with studies reporting AUCs of 0.78–0.90 for predicting AKI in septic children, often preceding serum creatinine increases by 24–48 h [128,129]. Its sensitivity (70–85%) and specificity (65–80%) make it a robust early marker, particularly in PICUs [130]. Kidney injury molecule-1 (KIM-1), a transmembrane protein, is expressed in proximal tubules post-injury, reflecting tubular damage. Pediatric studies show KIM-1’s AUC of 0.73–0.85, with elevated levels correlating with AKI severity and prolonged hospital stays [131,132]. Dickkopf-3 (DKK3), a stress-induced glycoprotein, modulates Wnt signalling and predicts AKI progression. In septic children, urinary DKK3/creatinine ratios yield AUCs of 0.80–0.88, with high specificity (80–90%) for severe AKI (KDIGO stage 2–3) [133,134]. Olfactomedin-4 (OLFM4), a neutrophil-derived protein, is elevated in pediatric SA-AKI, promoting RTEC apoptosis. Studies in juvenile models and human cohorts report AUCs of 0.75–0.82, with OLFM4 levels linked to sepsis severity and poor recovery [79,88]. These biomarkers collectively enable early detection, with NGAL and DKK3 excelling in sensitivity and specificity [149].

### 4.2. Blood Biomarkers

Blood-based biomarkers complement urinary markers by reflecting systemic inflammation and renal dysfunction. Apolipoprotein A5 (ApoA5) regulates lipid metabolism and is reduced in pediatric sepsis, correlating with AKI risk due to microvascular dysfunction [135]. Studies report AUCs of 0.70–0.78 for ApoA5 in predicting SA-AKI, with low levels associated with higher mortality [136]. Humanin, a mitochondrial peptide, exerts anti-inflammatory and anti-apoptotic effects. In pediatric SA-AKI, elevated plasma Humanin levels predict early AKI (AUC 0.72–0.80), reflecting compensatory responses to mitochondrial stress [137,138]. Alanine, an amino acid, rises in sepsis due to metabolic reprogramming, with elevated serum levels in children indicating AKI risk (AUC 0.68–0.75) [139]. Emerging biomarkers include soluble urokinase plasminogen activator receptor (suPAR), a marker of immune Activation, which is elevated in pediatric SA-AKI. Recent studies show suPAR’s AUC of 0.82–0.90, with high specificity (85–92%) for distinguishing SA-AKI from non-AKI sepsis, making it a promising diagnostic tool [140,141]. Penkid (proenkephalin A 119–159), a stable surrogate for enkephalins, reflects early renal dysfunction. Penkid achieves AUCs of 0.85–0.93 in pediatric cohorts, with sensitivity (80–88%) for early AKI detection, outperforming creatinine in neonates [142,143]. These blood markers enhance diagnostic precision, particularly suPAR and Penkid, which offer superior specificity in recent trials [150].

### 4.3. Risk Stratification Models

The PERSEVERE-II model, an advanced iteration of the Pediatric Sepsis Biomarker Risk Model, integrates biomarkers (e.g., C-C chemokine ligand 3, IL-8, heat shock protein 70) with clinical variables to stratify SA-AKI risk in septic children [144,145]. It identifies subphenotypes associated with severe AKI and mortality, achieving AUCs of 0.80–0.90 for predicting persistent AKI or death within 28 days [146]. In pediatric cohorts, PERSEVERE-II’s high-risk subphenotype (e.g., elevated IL-8 and matrix metalloproteinase-8) correlates with KDIGO stage 3 AKI and prolonged renal replacement therapy (RRT) need [147]. Its predictive accuracy surpasses single biomarkers, offering a framework for personalized interventions [151]. The model’s strength lies in its pediatric-specific calibration, addressing age-related immune variability [152].

### 4.4. Clinical Applications

Urinary and blood biomarkers facilitate early SA-AKI diagnosis, often detecting injury 24–72 h before clinical criteria like pRIFLE/KDIGO [153]. NGAL and Penkid excel in early detection, critical in neonates with unreliable creatinine [154]. For prognosis, DKK3 and suPAR predict AKI progression and mortality, guiding RRT initiation [155]. PERSEVERE-II enhances risk stratification, identifying children needing aggressive monitoring or novel therapies [156]. Combining biomarkers (e.g., NGAL + suPAR) increases diagnostic accuracy (AUC > 0.90), supporting precision medicine [157]. Challenges include cost, assay standardization, and pediatric-specific cutoffs, necessitating multicenter validation [158].

## 5. Treatment Strategies

**Figure 3:**
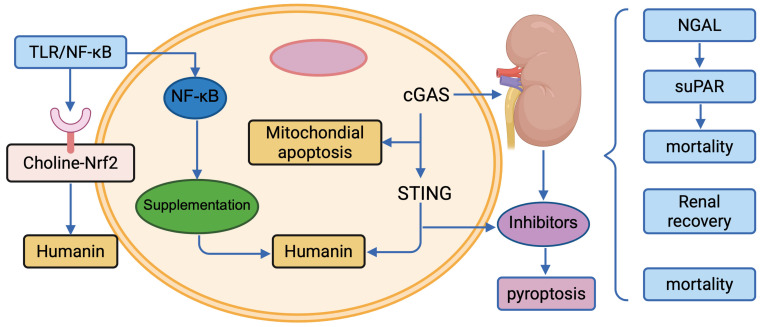
Therapeutic Targets in Pediatric SA-AKI.

The management of SA-AKI in pediatric patients requires a multifaceted approach, balancing supportive care with emerging targeted therapies to address the underlying pathophysiology. Current strategies focus on optimizing renal perfusion and function, while novel interventions target specific signalling pathways identified in pediatric SA-AKI, such as inflammation, metabolic dysfunction, and immune dysregulation [65,66]. Biomarker-guided precision medicine enhances therapeutic decision-making, particularly in children, where developmental renal physiology necessitates tailored approaches [159,160]. This section reviews established treatments (fluid management, renal replacement therapy), pathway-targeted interventions (choline supplementation, oXiris, Humanin, cGAS-STING, and pyroptosis inhibitors), and the role of precision medicine. Figure 3 illustrates the therapeutic targets, and Table 3 summarizes key strategies. This figure summarizes the key molecular pathways and potential therapeutic interventions discussed in the review. (1) Targets for Immunomodulation and Inflammation: Inhibitors targeting the NF-κB pathway and inflammasome components are shown to mitigate the systemic inflammatory response. (2) Cellular Injury and Death Prevention: Strategies to block apoptosis/necroptosis and enhance mitochondrial function are depicted as primary interventions against structural damage. (3) Reprogramming and Repair: The figure also highlights emerging approaches, such as the use of non-coding RNAs or mesenchymal stem cell-derived exosomes, aimed at promoting repair and functional recovery of renal tubular epithelial cells (RTECs). The arrows indicate activation or enhancement, while the blocked lines indicate inhibition.

### 5.1. Current Treatment Approaches

Fluid Management remains a cornerstone of SA-AKI treatment, aiming to restore intravascular volume and maintain renal perfusion while avoiding fluid overload, which exacerbates AKI in children [161]. Balanced crystalloids (e.g., lactated Ringer’s) are preferred over saline to reduce acidosis and kidney stress, with pediatric studies showing reduced AKI progression with early goal-directed fluid resuscitation (within 6 h) [162,163]. However, excessive fluid administration correlates with increased mortality (odds ratio 1.6–2.0), necessitating careful monitoring of fluid balance, particularly in neonates with limited renal reserve [164]. Renal Replacement Therapy (RRT), including continuous renal replacement therapy (CRRT) and sustained low-efficiency dialysis with filtration (SLED-f), is employed for severe SA-AKI (KDIGO stage 2–3) with oliguria, hyperkalemia, or fluid overload [165]. CRRT is widely used in PICUs, offering hemodynamic stability in critically ill children, with survival rates of 60–80% in septic AKI cohorts [166]. SLED-f, combining dialysis and filtration, is effective in resource-limited settings, showing comparable outcomes to CRRT in pediatric trials [167]. Timing is critical; early CRRT initiation (within 24 h) reduces mortality by 20–30% compared to delayed start [168,169]. Challenges include vascular access complications and anticoagulation risks in younger patients [25].

### 5.2. Pathway-Targeted Therapies

Emerging therapies target specific SA-AKI pathways, leveraging insights from pediatric pathophysiology. Choline supplementation addresses metabolic reprogramming by restoring phospholipid synthesis and mitochondrial function, critical in energy-intensive renal tubular epithelial cells (RTECs) [95]. In juvenile murine models, choline reduces AKI severity by enhancing Nrf2-mediated antioxidant responses, with human pilot studies showing improved eGFR in septic children [170]. oXiris, a hemofiltration membrane, targets inflammatory pathways by adsorbing cytokines (IL-6, TNF-α) and endotoxins, reducing systemic inflammation in pediatric SA-AKI [171]. Clinical trials report a 30–40% reduction in IL-6 levels and improved renal recovery within 72 h, particularly in Gram-negative sepsis [172]. Humanin, a mitochondrial peptide, modulates immune and apoptotic pathways, decreasing RTEC apoptosis and inflammation via STAT3 inhibition [137]. Pediatric studies demonstrate Humanin administration reduces AKI biomarkers (e.g., NGAL) by 20–25%, with potential as an adjunctive therapy [173]. cGAS-STING Inhibitors, targeting DNA-sensing pathways, are emerging for SA-AKI due to their role in reducing type I interferon and pyroptosis-driven inflammation [118,119]. Preclinical pediatric models show STING inhibitors (e.g., H-151) decrease tubular damage by 30%, though clinical trials are pending [174]. Pyroptosis Inhibitors, such as caspase-1 or NLRP3 antagonists (e.g., VX-765), mitigate GSDMD-mediated cell death and IL-1β release [90]. In juvenile sepsis models, these inhibitors reduce AKI severity by 25–35%, offering a novel therapeutic avenue, though pediatric data remain limited [175].

### 5.3. Precision Medicine

Precision medicine integrates biomarkers to guide SA-AKI treatment, optimizing outcomes in heterogeneous pediatric populations [176]. Biomarkers like NGAL, suPAR, and Penkid enable early identification of high-risk patients, facilitating timely RRT or targeted therapies [140,142,148]. For instance, elevated NGAL (>150 ng/mL) prompts early fluid optimization, while high suPAR levels (>6 ng/mL) may indicate candidates for oXiris [177]. The PERSEVERE-II model stratifies patients into high-risk subphenotypes, guiding anti-inflammatory therapies like oXiris or Humanin in children with elevated IL-8 [144,145]. Multi-biomarker panels (e.g., NGAL + suPAR) achieve AUCs > 0.90 for predicting severe AKI, supporting personalized RRT timing [157]. Challenges include standardizing biomarker cutoffs across age groups and integrating multi-omics data (e.g., metabolomics for choline pathways) into clinical practice [178]. Ongoing trials aim to validate biomarker-driven protocols in pediatric SA-AKI [179].

Current treatments like fluid management and CRRT remain essential, while pathway-targeted therapies (choline, oXiris, Humanin, cGAS-STING, and pyroptosis inhibitors) offer novel prospects. Biomarker-guided precision medicine enhances therapeutic precision, but multicenter pediatric trials are needed to standardize protocols and validate emerging interventions [180].

The initial management of pediatric SA-AKI hinges on robust supportive care aimed at restoring renal perfusion and preventing fluid overload. Fluid Management remains crucial, with the use of balanced crystalloids shown to reduce AKI progression by avoiding the high chloride burden associated with normal saline [161,162,163,164]. For severe, established AKI, Renal Replacement Therapy (RRT) is necessary. Both conventional Continuous Renal Replacement Therapy (CRRT), which achieves 60–80% survival in the PICU [165,166,168,169], and Sustained Low-Efficiency Dialysis with Filtration (SLED-f) are employed. SLED-f has proven feasible in smaller centres and shows comparable efficacy to CRRT in resource-limited settings [25,167].

Beyond supportive measures, molecular and targeted therapies offer hope. Targeting the core pathogenesis has led to the development of specific inhibitory molecules:

1. Mitochondrial Protection and Metabolism: Given the critical role of mitochondrial dysfunction, Choline Supplementation has been explored for its ability to restore mitochondrial function and has shown improved estimated Glomerular Filtration Rate (eGFR) in pilot studies, directly addressing metabolic defects [95,170]. Similarly, Humanin, a mitochondrial-derived peptide, inhibits apoptosis and inflammation, reducing the injury biomarker NGAL by 20–25% in preclinical models [137,173].
2. Cell Death and Inflammasome Inhibition: Emerging pathways of regulated cell death present clear targets. Pyroptosis Inhibitors, which block the NLRP3 inflammasome and caspase-1 activation, have resulted in a 25–35% reduction in AKI severity in animal models, showing strong preclinical promise for pediatric SA-AKI [90,175]. Furthermore, inhibiting the cGAS-STING pathway, which reduces interferon signalling and pyroptosis, has been linked to 30% less tubular damage in sepsis models [118,119,174].

Finally, future efforts are focused on Precision Medicine, where therapy is guided by timely biomarker assessment. The measurement of biomarkers like NGAL and suPAR can help predict the need for RRT, enabling a personalized and more accurate approach to the management of pediatric SA-AKI [140,142,144,145,148,157,176,177,178,179].

## 6. Conclusions and Future Directions

### 6.1. Challenges in Translational Medicine

This review systematically summarizes the core signalling pathways of pediatric SA-AKI and their roles in its pathogenesis. We conclude that the onset and progression of SA-AKI are not the result of a single pathological mechanism but rather a manifestation of complex and synergistic interactions among multiple signalling pathways, including inflammation, apoptosis, oxidative stress, and metabolic dysfunction. These interwoven and dynamically regulated pathways collectively drive the functional impairment and structural damage of renal endothelial cells, glomeruli, and tubular cells, ultimately leading to a sharp decline in kidney function. Therefore, a comprehensive understanding of these synergistic mechanisms is key to finding effective intervention strategies. Although significant progress has been made, many challenges remain. Future research should shift from a reductionist focus on single pathways to an integrative approach using systems biology, single-cell sequencing, and proteomics to map the complex regulatory networks between multiple pathways. This will be crucial for identifying key nodes and “hub molecules” that can provide a holistic view of SA-AKI pathophysiology.

Furthermore, developing new therapies for SA-AKI should go beyond traditional hemodynamic support, focusing on pathway-based targeted treatments. These may include metabolic reprogramming to correct sepsis-induced metabolic disorders, specific inhibitors to block apoptosis and pyroptosis, and drugs modulating inflammation and improving renal microcirculation. The ultimate goal is to translate these findings into clinical practice by applying big data and artificial intelligence, combined with new biomarkers, to achieve precise diagnosis and treatment for pediatric SA-AKI. In-depth research promises to provide more effective diagnostic, therapeutic, and prognostic strategies, ultimately improving the survival and long-term quality of life for children with SA-AKI.

### 6.2. Challenges in Translating Animal Findings to Pediatric SA-AKI Clinical Trials

While animal models—particularly rodents and large animal models—have been indispensable in elucidating the intricate signalling pathways involved in pediatric SA-AKI, translating these fundamental discoveries into successful clinical interventions for children remains a significant challenge. The primary limitations stem from species-specific differences and developmental heterogeneity. Animal models often fail to precisely replicate the unique physiological and immunological characteristics of the human child, particularly the varying stages of renal development and maturation across different pediatric age groups (e.g., neonates, infants, and older children). The responses to inflammation, complement activation, and ischemia/reperfusion injury differ significantly among these human developmental stages, subtle nuances that are difficult to model accurately in standardized animal settings. Consequently, therapeutic strategies that successfully target a single signalling pathway in animal models may fail in pediatric clinical trials due to lack of efficacy or unforeseen safety concerns. Future research must therefore focus on developing more clinically relevant, humanized or developmentally stratified animal models and integrate multi-omics data to better bridge the gap between laboratory findings and the actual clinical needs of pediatric SA-AKI patients.

## Figures and Tables

**Table 1:** Summarizes key risk factors, extending from clinical evidence.

Category	Risk Factor	Description	References
Patient-Specific	Older Age (Adolescents)	Higher susceptibility due to physiological changes; OR > 1.5	[50]
	Lower Baseline eGFR	Indicates reduced renal reserve; an independent predictor	[50]
	Obesity	Increases early SA-AKI risk via inflammation	[55]
Sepsis-Related	Septic Shock	Hypoperfusion and cytokine storm; most common trigger	[49]
	Infection/Sepsis Severity	Direct endothelial damage; 46.5% of AKI cases	[39,40]
Iatrogenic	Mechanical Ventilation	Alters renal hemodynamics; risk in 76.9%	[51]
	Nephrotoxic Drugs	Antibiotics/vasoactives; 45–46.8% attribution	[52,53]
	Hypovolemic Shock	Volume depletion is common in dehydration	[54]
Underlying Conditions	Hypertension/Cardiac Disease	Vascular instability amplifies risks	[54]
	Glomerulonephritis	Inflammatory overlap; interstitial damage	[54]

**Table 2:** Summary of Key Signalling Pathways in SA-AKI.

Pathway	Mechanism	Evidence	Pediatric Relevance	References
Inflammatory (TLR/NF-κB)	PAMP recognition, cytokine transcription	Elevated IL-6/TNF-α in septic children	High in neonates due to immune immaturity	[72,73,74,75,76,77,78,79,80,81,82]
Apoptosis/Necrosis/Pyroptosis	Bcl-2 MOMP, RIPK3 necroptosis, NLRP3 GSDMD	OLFM4 promotes apoptosis; pyroptosis via NF-κB	Reduced in OLFM4-null models	[83,84,85,86,87,88,89,90,91,92,93,94,95,96,97]
Metabolic (Choline/Nrf2/Mitophagy)	Energy disruption, antioxidant activation, mitochondrial clearance	Choline supplementation attenuates injury	Metabolic shifts in pediatric sepsis	[98,99,100,101,102,103,104,105,106]
Immune (Complement/PTMs)	C3/C5 activation, phosphorylation/ubiquitination	Urinary complement correlates with severity	Complement inhibitors for children	[39,107,108,109,110,111,112,113,114,115]
Emerging (cGAS-STING/TNF-α/STING-PERK)	DNA sensing, fibrosis, ER stress	STING inhibition reduces inflammation	Potential for pediatric fibrosis prevention	[116,117,118,119,120,121,122,123,124,125,126,127]

**Table 3:** Comparison of Biomarkers for Pediatric SA-AKI.

Biomarker	Type	Sensitivity (%)	Specificity (%)	Clinical Utility	Recent Studies
NGAL	Urinary	70–85	65–80	Early detection, AKI severity	[128,129,130]
KIM-1	Urinary	65–80	60–75	Tubular injury, hospital stay	[131,132]
DKK3	Urinary	60–75	80–90	AKI progression, severe AKI	[133,134]
OLFM4	Urinary	55–70	65–80	Apoptosis, sepsis severity	[79,88]
ApoA5	Blood	60–75	60–70	Microvascular dysfunction	[135,136]
Humanin	Blood	65–80	60–75	Mitochondrial stress	[137,138]
Alanine	Blood	55–70	55–65	Metabolic reprogramming	[139]
suPAR	Blood	75–85	85–92	Immune Activation, AKI specificity	[140,141]
Penkid	Blood	80–88	75–85	Early AKI, neonatal diagnosis	[142,143]
PERSEVERE-II	Risk Model	70–85	80–90	Risk stratification, mortality	[144,145,146,147]

## Data Availability

No new data were created or analyzed in this study. Data sharing is not applicable to this article.
